# The role of conflict processing mechanism in deception responses

**DOI:** 10.1038/s41598-022-21569-7

**Published:** 2022-10-31

**Authors:** Jintao Wu, Jie Huang, Jiaxuan Li, Xianglin Chen, Yi Xiao

**Affiliations:** 1 Beijing Machine and Equipment Institute, Beijing, 100854 China; 2 National Key Laboratory of Human Factors Engineering, China Astronaut Research and Training Center, Beijing, 100094 China; 3 Beijing HuiLongGuan Hospital, Peking University HuiLongGuan Clinical Medical School, Beijing, 100096 China; 4 Beijing Institute of Information Technology, Beijing, 100094 China

**Keywords:** Human behaviour, Cognitive neuroscience

## Abstract

A considerable number of studies have described the potential neural mechanism of deception, but most deception studies have relied upon deception from experimental supervisor instruction. Experimental control (participants follow instructions to deceive without any risk) means that the deception occurs in a way that does not come close to the real deception. In the current study, a neural imaging experiment on deception closer to the real deception was conducted. Event-related potential (ERP) and event-related spectral perturbation (ERSP) techniques were used to explore the neural mechanism of deception. The results showed that deceptive response evoked larger medial-frontal negativity (MFN) and smaller response-locked positivity (RLP) than truthful response. We interpret these findings to indicate that conflict detection and emotional processing are associated with deception. In addition, magnitudes of alpha and beta oscillations after the deceptive response were significantly smaller than those after the truthful response, demonstrating that deception is associated with neural oscillations reflecting conflict adjustment. The results comprehensively characterized the physiological properties of the brain oscillations elicited by a deceptive response and provided a theoretical foundation for detection in practical applications.

## Introduction

The importance of detecting deception in criminal investigations, counterterrorism inquiries and security reviews is evident. Although extensive research has been conducted on the neural basis of deception^1,2^, the mechanism of how the brain is involved in deception processing is still unclear. In recent years, the rapid development of brain imaging techniques and analysis techniques such as event-related potential (ERP) and functional magnetic resonance imaging (fMRI) have made it possible to accurately record brain activity during deception.

Deception processing involves several dynamic stages, during which executive functioning plays an important role^3^, including working memory, conflict control, response inhibition, emotional processing and task switching. ERPs were used to study the time course of complex changes in brain activity during deception. For example, deceptive responses evoked smaller stimulus-locked later positive component (LPC) than truthful responses^4^. P300-based Guilty Knowledge(GKT) or Concealed Information Test (CIT)^5–8^ were also used to detect concealed information. GKT is nowadays commonly referred to as the CIT. Essentially CIT does not detect deception itself, but mainly detects memory related to crime or concealed information^9,10^. Because of relevant memories, the crime or concealed information was only sensitive to the guilty examinees. The main assumption of the CIT is that the familiar stimuli would induce different responses when occurred in the context of many homogeneous unfamiliar stimuli. Because the familiar stimuli are similar to the target stimuli in the conventional “oddball” test compared with the other unfamiliar stimuli with high stimuli frequency, the ERP (e.g. P300) could be observed from the responses of guilty examinees^11^. During the test, memories of crimes or concealed information were stored in the brain, and the ERP results were the same regardless of whether the subjects were lying or telling the truth. In order to induce more obvious ERP characteristics, so as to identify criminal suspects more accurately and efficiently, the subjects are usually asked to respond to the presented stimuli in the process of testing, thereby introducing deception into the decision-making process. In other words, in deception-related studies, memory is detected when the stimulus is presented, and deceptive response per se is detected when responding to the stimulus. Abe et al. thought that in the study of deception, both the memory under specific conditions and the deceptive response per se should be taken into account^9^. Therefore, the ERP components concerned in the research of deception detection involve not only stimulus-locked but also response-locked.

The medial-frontal negativity (MFN) is the response-locked component associated with deception. Johnson et al. conducted a series of studies to explore the brain activity patterns in deceptive response. Their results showed that deceptive responses elicited more negative MFN than truthful responses, and that MFN was thought to be related to response monitoring and conflict detection^4,12–14^. MFN was supposed to reflect the conflict between the deceptive response that has been performed (incompatible with the truth) and the truthful response that is still activating after the response (compatible with the truth)^15^. The component was originally found in error responses in which the stimuli elicited conflicting response tendencies^12,16,17^, it is therefore named error-related negativity (ERN) or Ne (error negativity). Later, other studies also found a similar, albeit with a smaller negative component in correct responses^18–21^. Considering that the topographical distribution of the MFN is very similar to that of ERN, which is usually observed during responses to unintended consciously recognized errors^22^, as well as the conflict characteristics of deception (conflict between an automatically activated truthful response and a controlled deceptive response) that are similar to error detection, we assumed that the conflict detection mechanism is involved in deception processing. Therefore, deception processing may be similar to error detection^23–25^. According to this assumption, deliberately deceptive claims could be classified by the brain as incompatible behavior or an “error” because it goes against the trend observed for a truthful response^26^.

Deception processing not only affects the phase-locked ERPs in the time domain but also may affect the nonphase-locked event-related synchronization (ERS)/event-related desynchronization (ERD) in the time–frequency domain. Some research has shown that the alpha oscillations after the incongruent condition are significantly lower than those after the congruent condition in the Stroop task, which indicated that the brain adjusts the attention resource in real time according to the conflict information^27^. When the brain is in the attention state, alpha-ERS in the frontal-parietal region is lower than that in the resting state. It shows that alpha oscillations are negatively correlated with the brain activation state, and the stronger the oscillations (ERS enhancement), the lower is the activation. In contrast, the attenuation of oscillations (ERD enhancement) reflects heightened brain activation^28,29^. Therefore, the increase in alpha-ERD during deception may be related to the increase in the attention and cognitive load^30,31^. Moreover, decreased beta oscillations were observed to be associated with a deception response^32,33^, which is a reflection of reward evaluation after deception. Reduced beta oscillations were also observed during the conflict task, suggesting that beta oscillations are linked to conflict inhibition^34^. These studies may reflect that deception with conflict characteristics can affect beta oscillations.

Previous deception studies have investigated various types of deception behaviors with different tasks and stimuli, such as answering questions related to autobiographical semantic memory^35,36^, committing a mock crime and then giving deceptive or honest answers in the following test^32,37,38^, and utilizing deception to defeat an opponent in a simulated game^26,39–41^. However, the results were not consistent, which directly led to the inconsistency of the theoretical basis of deception. A main reason for the difficulty is the extreme lack of ecological validity for deceptive research^42^. In most previous deception studies, participants were instructed by the experimenter to make deceptive or truthful responses under corresponding conditions, and the responses in the experiment were monitored by the experimenter. Since the participants are aware that each of their deceptions is detected by the experimenter, their psychological state is different from that during real deception. Although some studies used spontaneous deception tasks, participants clearly know that even if they are deceptive, there will be no additional rewards or punishment, it is difficult to induce strong emotional and psychological conflicts regarding deception^43^.

The purpose of this study is to reveal the neural mechanisms of deception through experiment that more closely resemble real deception. We focused on the deceptive response per se rather than memory recognition of concealed information, so the response-locked EEG activity was analyzed. An experimental design similar to CIT was used, but because we did not focus on stimulus-locked ERP (e.g. P300), and in order to obtain a comparable number of deceptive and truthful responses, the presentation frequency of different types of stimuli was set to the same. To make the deception responses more realistic, the person instructing the participant to deceive and the person testing the participant were assigned to different people. From the participant's point of view, the instructor and investigators were unaware of each other's existence. In addition, the deception task involved real deception events, and stimulus materials were encoded by real events.

Due to the different cognitive processes and cognitive loads of deception and truth-telling, deceptive and truthful responses are assumed to lead to different cortical activation patterns. We hypothesized that deceptive response would evoke a larger MFN than truthful response, and induce smaller alpha and beta band oscillations.

## Results

### Behavioral results

**Figure 1:**
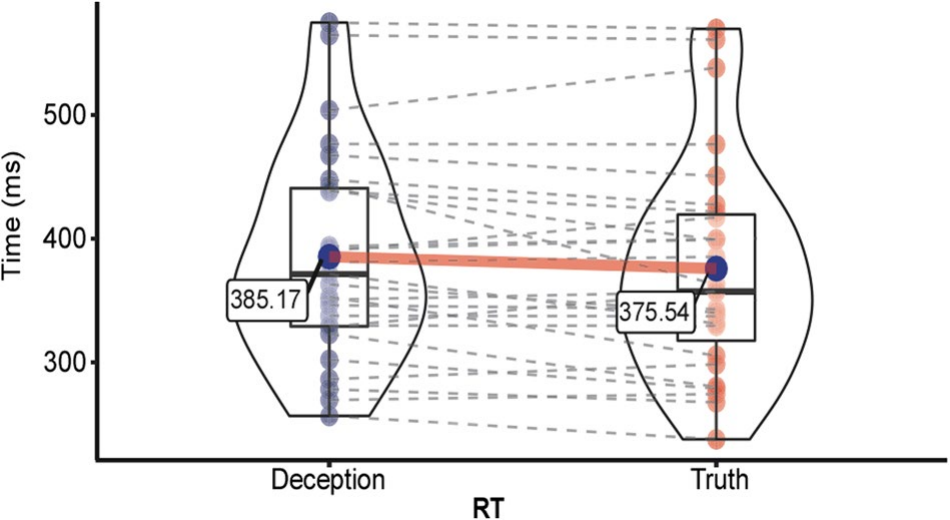
Average response time of deceptive and truthful responses. Paired t-test results showed marginal significance (p = 0.059).

As shown in Fig. 1, the RTs of deceptive responses (385.17 ± 84.47 ms) were longer than the RTs of truthful responses (375.54 ± 88.09 ms), and the difference was marginally significant (t(26) = 1.976, p = 0.059).

### Time domain analysis

**Figure 2:**
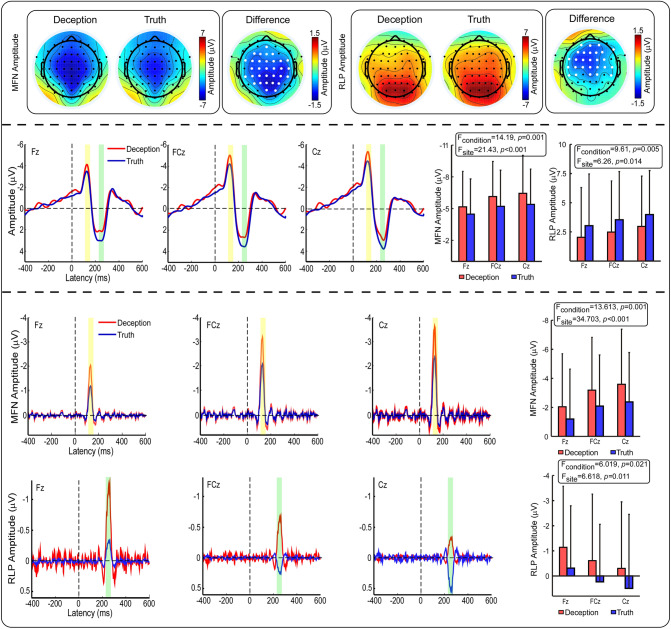
Comparison of ERPs between the deception and truth conditions. Top panel: scalp topographies of MFN and RLP waves are displayed at their peak latencies (MFN: 126 ms; RLP: 264 ms). The enlarged white dots indicate electrodes with significant differences. Middle panel: group average waves at three fronto-central electrodes (Fz, FCz and Cz) are shown. Compared with the truthful response (blue), the deceptive response (red) had a significantly larger MFN and smaller RLP. Bottom panel: PCA results of MFN and RLP. There was a significant increase in MFN and a significant decrease in RLP in the deceptive response compared to the truthful response. The light yellow bar and light green bar in the middle panel represent the time range of MFN (110–150 ms) and RLP (240–280 ms), respectively.

The average response-locked ERP waveforms at Fz, FCz and Cz are shown in Fig. 2.

For MFN, repeated measures ANOVA revealed a main effect of condition (F (1, 26) = 14.19, p < 0.001, η_p_^2^ = 0.35) with a greater amplitude for deceptive responses than for truthful responses. A significant main effect of site was also found (F (1.199, 31.177) = 21.43, p < 0.001, η_p_^2^ = 0.45), with a more negative amplitude at Cz than at Fz and FCz. No further interactions reached significance (F (1.174, 30.514) = 1.41, p > 0.05, η_p_^2^ = 0.05). Similarly, the MFN component score also showed that there were significant condition main effect (F (1, 26) = 13.613, p < 0.001, η_p_^2^ = 0.344) and site main effect (F (1.315, 34.196) = 34.703, p < 0.001, η_p_^2^ = 0.572) in MFN amplitude, but the interaction effect was not significant (F (1.224, 31.824) = 1.501, p > 0.05, η_p_^2^ = 0.055).

For RLP, the condition main effect highlighted that the amplitude for deceptive responses was smaller than that for truthful responses (F (1, 26) = 9.61, p < 0.05, η_p_^2^ = 0.27). A significant main effect of site (F (1.189, 30.919) = 6.26, p < 0.05; η_p_^2^ = 0.19) indicated that the amplitude was more positive at Cz than at Fz and FCz. However, the interaction effect of the two factors was not found (F (1.265, 32.882) = 0.065, p > 0.05; η_p_^2^ = 0.003). The same results were also found in the RLP component score, the main effect of condition (F (1, 26) = 6.019, p < 0.05, η_p_^2^ = 0.188) and the main effect of site (F (1.23, 31.982) = 6.618, p < 0.05, η_p_^2^ = 0.203) were statistically significant, but their interaction did not reach significance (F (1.261, 32.775) = 0.04, p > 0.05, η_p_^2^ = 0.002).

### Source localization

**Figure 3:**
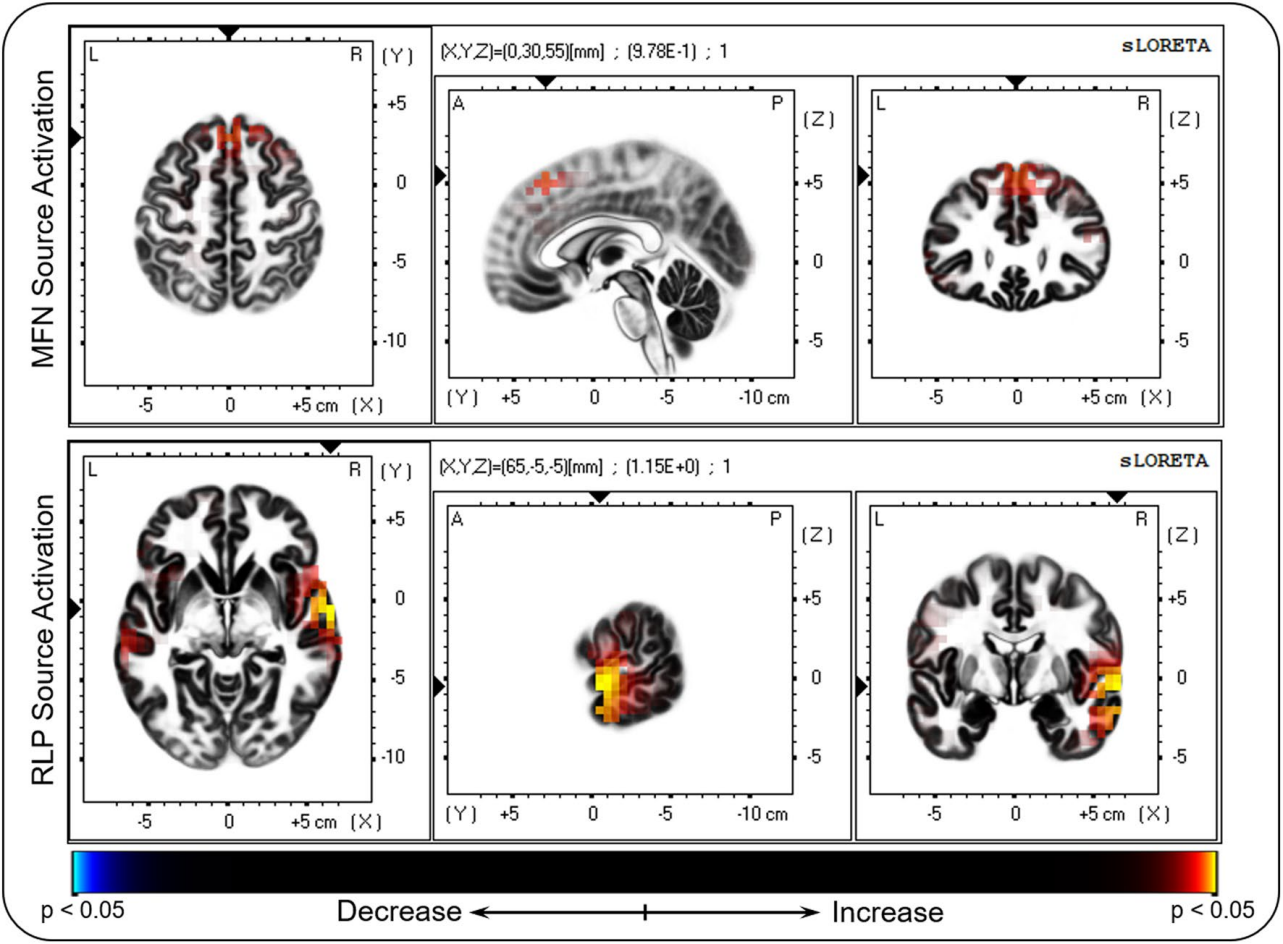
Source activation differences between the truth and deception conditions at MFN and RLP time windows. Top panel: superior frontal gyrus. Bottom panel: middle temporal gyrus and superior temporal gyrus. Red and yellow colors indicate increased activity in the deception condition.

Source analysis via sLORETA revealed differential activities in intervals of 112–142 ms and 186–278 ms following truthful and deceptive responses (t = 0.877, p < 0.001; t = 0.882, p < 0.001) (see Fig. 3). The source imaging showed that MFN source densities of deceptive responses were mainly enhanced in the superior frontal gyrus (SFG, BA 8). RLP source densities of deceptive responses were more pronounced in the middle temporal gyrus (MTG, BA 21) and superior temporal gyrus (STG, BA 22) than in truthful responses.

### Time–frequency domain analysis

**Figure 4:**
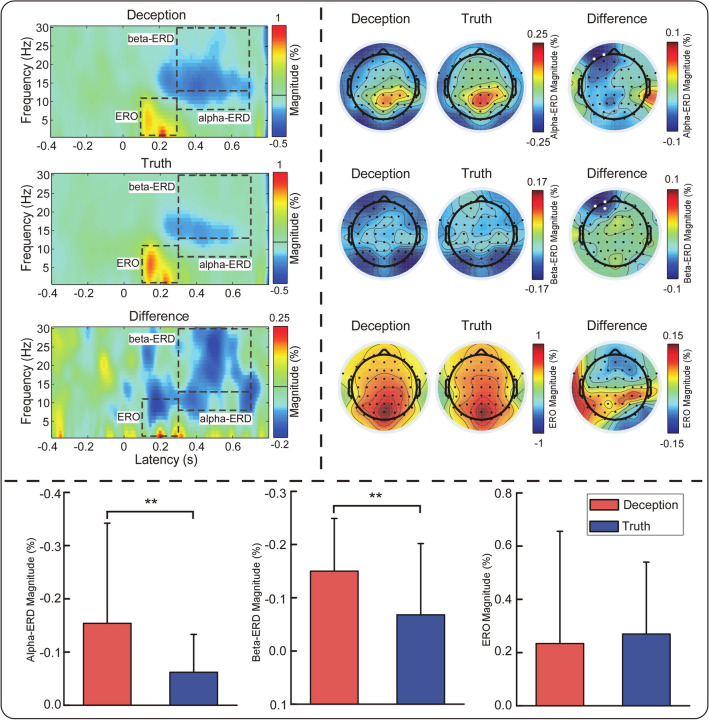
Comparison of time–frequency distributions (TFDs) between the deception and truth conditions. Upper-left panel: displayed signals showed EEG oscillations at the AF7 electrode. TFDs contained a phase-locked response (ERO: 100–300 ms, 1–11 Hz) and two nonphase-locked responses (alpha-ERD: 300–700 ms, 8–13 Hz; beta-ERD: 300–700 ms, 13–30 Hz), highlighted by the black dashed lines. Upper-right panel: scalp topographies of the response magnitudes in the deception and truth conditions. The significant differences are represented by enlarged white dots. Bottom panel: alpha-ERD and beta-ERD magnitudes (measured at the AF7 electrode) in the deception condition were significantly larger than those in the truth condition.

Responses induced an obvious phase-locked ERO (100–300 ms and 1–11 Hz) and two nonphase-locked ERDs (alpha-ERD, 300–700 ms and 8–13 Hz; beta-ERD, 300–700 ms and 13–30 Hz) (see Fig. 4). The amplitude of alpha-ERD for the deceptive response was significantly greater than that for the truthful response (− 0.154 ± 0.188 vs − 0.062 ± 0.7, p < 0.05, t = − 2.587). In addition, the amplitude of beta-ERD was significantly larger for a deceptive response than for a truthful response (− 0.150 ± 0.099 vs − 0.068 ± 0.134, p < 0.05, t = − 2.454). However, the evoked event-related oscillations (ERO) magnitude was not significantly different between the two conditions (0.234 ± 0.422 vs 0.270 ± 0.365, p > 0.05, t = − 0.534).

## Discussion

In the present study, ERP and ERSP techniques were used to measure the electrophysiological indices of deception through a deception task closer to real deception.

Our results revealed three marked differences between truthful and deceptive responses. Behaviorally, the RTs for deceptive responses were longer than those for truthful responses. In addition, in the time domain, relative to truthful responses, deceptive responses induced significantly larger MFN and smaller RLP. Finally, in the time–frequency domain, deceptive responses decreased the alpha and beta oscillation magnitudes. These results suggest that brain activities were enhanced when individuals performed deceptive responses.

The behavioral data revealed that RTs for deceptive responses were longer than those for truthful responses, which showed a marginal effect. In line with previous studies^9,38,44–46^, prolonged RTs indicate that deception-related cognitive processes are more complex than truth-related cognitive processes. Hence, when making deceptive responses, individuals encounter more cognitive conflicts and cognitive loads, which may lead to requiring additional time to process^33^.

In the time domain, the MFN analysis revealed a significantly more negative amplitude for deception responses compared to that for truth responses. This finding was in line with previous studies^9,38,44,47^, which indicated that MFN is associated with response monitoring and conflict detection^13^. This finding also supported the behavioral result that conflict detection increases the cognitive load. In the present research, the source generators of the difference in MFN were located in the SFG, which was somewhat different from other studies that indicated the anterior cingulate cortex (ACC)^9,12,19,48^. In fact, fMRI studies have found that increased activities in the SFG and ACC were associated with deception^49,50^. In addition, Rushworth et al. found that the specific brain area responsible for conflict monitoring or error monitoring is the SFG rather than the ACC^51^. They argued that because the ACC and SFG are very close in proximity, the distinction between the two regions is not very clear. Given the evidence that MFN was produced in or near the ACC (e.g., SFG)^12,13,19^, the current result is essentially consistent with those of fMRI studies, indicating that the SFG may be involved in conflict detection. In brief, a conflict between an automatically activated truthful response and a controlled deceptive response activated the SFG area, and the enhanced MFN was the reflection of conflict detection.

The current study also found a significant deception effect on RLP, with a smaller amplitude for deceptive responses compared to truthful responses. RLP generators were located in the MTG and STG. Previous fMRI studies have shown that the MTG and STG are more active in deception than in truth-telling^35,52–54^. Our results support these studies finding that the MTG and STG are associated with deception. Furthermore, studies have found that MTG and STG activities are associated with emotional processing^55–57^. Therefore, the RLP in our results may be related to emotional processing in deceptive responses. In fact, it is known that moral conflict is present in the process of deception, and people inevitably produce emotional fluctuations, such as tension and fear^35,58,59^. The dual-competition model proposed by some researchers suggests that emotional processing will occupy part of the resources and inhibit conflict resolution^60–62^. The conflict monitoring model proposed by Botvinick et al. holds that when individuals monitor conflict stimuli, top-down attention will be activated, and this mechanism can deal with conflicts through top-down control^63^. According to this model, emotional processing consumes or partially occupies the processing resources for top-down control, which weakens the conflict resolution. Our results can be explained by the two theoretical models, and a reduction in RLP is the reflection of emotional processing. In other words, negative emotions occur after deception, which activates the MTG and STG. Emotional processing partly occupies the cognitive resource for conflict detection, hence showing decreased RLP. To our knowledge, previous deception studies have rarely found obvious RLP, which may be because the emotion fluctuations involved in deception cannot be fully elicited by simulated experiments^43^, resulting in the absence of RLP.

There is also another possible interpretation for RLP. The MFN-RLP complex found in the current study is very similar to the ERN-Pe (error positivity) complex in error processing. While the ERN-Pe complex reflects the two stages of error processing, from the above ERP results, the MFN-RLP complex also reflects the two stages of deception processing. Considering that deception and error processing involve a common conflict detection neural mechanism and that MFN has functional significance similar to that of ERN, RLP may also have functional significance similar to that of Pe. Researchers have found that Pe amplitudes reflect subjective confidence and have suggested that Pe signals a confident response^64^. Therefore, similar to Pe, the pattern of RLP could also be interpreted as reflecting confidence. Deception is contrary to the concept of morality and may be related to lower levels of confidence in one's deceptive responses. This explanation is consistent with the current RLP results, although we did not acquire any confidence ratings.

Furthermore, interestingly, the waveform, latency and scalp topography of RLP are similar to P300. The P300 is a positive component peaking between 300 to 600 ms post-stimulus, with greatest amplitude at centroparietal scalp sites^65,66^. More, even if there appears to be a consensus that P300 has multiple neural generators, the brain sources found in present study are compatible with previous findings in the literature indicating temporal-parietal junction and adjacent areas clusters^67^. Previous studies have found that P300 component also appear after response-locking EEG and is thought to vary with risk level, motivational and emotional outcomes^68,69^. As mentioned earlier, deception in this study is also related to risk, motivation, and emotion. Considering the above factors, it is reasonable to assume that the two components, RLP and P300, may be different manifestations of the same physiological and functional system.

In the time–frequency domain, compared with truthful responses, deceptive responses elicited an increased alpha-ERD and beta-ERD in the left prefrontal cortex, whereas they elicited a comparable amplitude of power in the phase-locked ERP. Previous studies confirmed that the alpha band was sensitive to deception^30,31^. It has been found that the increase in alpha band power indicates that the brain is spontaneously unloading or idling^70,71^, and the power reduction indicates that the brain is in a high cognitive load state. The increased alpha-ERD in the deceptive response in the present study may reflect the real-time psychological adjustment to the conflicts. Similarly, the decrease in the beta oscillations for deceptive responses was found in previous research^33^. A separate study also found that beta-band functional connections between the frontal and parietal regions were reduced during deception^32^, which suggested that beta-band modulation during deception is related to the mechanism of conflict inhibition. Zavala et al. found that beta oscillations reflect the coordination of neural activity during response inhibition^34^. Consistent with these results, our study suggested that decreased beta oscillations in deception may be related to the inhibition of truthful response in the conflict, which arises from automatically activated truthful response and a controlled deceptive response. Considering the changes in alpha and beta band power, the results indicate that deception involves greater cognitive load and conflict adjustment than a truthful response. Moreover, no difference in evoked ERO power was found in different types of responses, which may be reconciled by the MFN and the RLP components with the contradictory changing trends. These findings provide further evidence regarding the conflict process of deception.

Taken together, our findings elucidated that when generating a deceptive response, cognitive and psychological (emotional) activities were enhanced. The neural activity changes found in this study can be used as neurophysiological features of deceptive processing, which perform executive function by gating deceptive information through top-down control. Our results theoretically illustrate our understanding of the potential neural mechanisms associated with top-down modulation of deceptive responses and, in a practical sense, help to improve the value of deception detection in various practical applications.

It is worth mentioning that our experimental paradigm seems to be similar to CIT. The existing research on deception includes not only the study of memory related to concealed information, but also the study of deception itself. As mentioned above, the CIT detects specific memories, not the deceptive response itself. In our experiment, deceptive responses were closely related to prior knowledge of experienced events, which seems to be similar to the interrogation used in the CIT. Apart from the fact that the frequencies of stimuli are not consistent with that of CIT, the biggest difference is that we study the deceptive response itself, so we only focus on the response-locked ERP and EEG. While the CIT mainly detects memory related to concealed information and focuses on the stimulus-locked ERP (e.g. P300). Therefore, the current research is different from CIT research.

Nevertheless, our study has several limitations. First, in our deception measurement paradigm, RLP elicited by deceptive responses have not been found in other deception studies, so the convergent validity of the new and old paradigms cannot be provided. This may be because our paradigm is closer to real deception than that used in the previous deception paradigm, thus leading to new findings. It would, however, be very helpful for future research to verify the results with paradigms using real deception. Second, despite our experimental design attempted to simulate an near-to-real-life scenario, the ecological validity is still insufficient as the deception is still being investigated in the laboratory. Third, the sample size was rather small, which might lower the generalisability of the findings. A larger study will be needed to validate these observations.

In summary, this study used ERP and ERSP techniques to explore the potential neural mechanisms of ecologically valid deception. Deception induces conflict detection and increases cognitive load, which is reflected by larger MFN in the early stage. During the postdeception conflict adjustment stage, negative emotional processing partly occupies cognitive resources, resulting in a decrease in RLP. Furthermore, alpha and beta oscillations are also involved in conflict adjustment, and the increased alpha-ERD and beta-ERD are the reflection of the top-down conflict adjustment. All results confirmed that deception generated psychological and cognitive conflict, which are reflected through brain responses.

## Methods

### Participants

Thirty-three healthy, right-handed male students from People’s Public Security University of China were recruited. All the subjects were between 21 and 25 years old (22.47 ± 0.74 years) and had normal or corrected-to-normal vision. The data of six volunteers were excluded: four because they did not exhibit deception and two because they had excessive artifacts. Finally, twenty-seven (17 males and 10 females) participants were included in the analysis. Written informed consent was acquired from all subjects prior to the study. The study was in accordance with the Declaration of Helsinki and was approved by the Ethics Committee of China Astronaut Research and Training Center.

### Task and procedure

Two days before the experiment, the participants were escorted to a laboratory by two teachers they had known. The two teachers first introduced themselves and then recounted the history and use of the laboratory. Finally, the volunteers were told they would take part in a confidential experiment in the laboratory, but they have to maintain secrecy about what they observed there and pretend they did not know the teachers or the place when asked by anyone. The subjects were also told that they would receive credit if they kept the secret until the end of the experiment.

In the deception research, the subjects were invited to participate in a survey on the current situation of scientific research in the school. The survey was conducted as a computerized test supervised by an official school authority with the implication that subjects would be punished for lying to the official school authority, and EEG data were recorded during the survey. Before the test, subjects did not know that the test content would be related to the previous confidential event.

The stimulus in the task consisted of three types of photos of people: probe stimuli (the photos of the two teachers mentioned above that required deceptive responses) and two types of nonprobe stimuli (photos of unknown people, which required "no" responses, and celebrities who are well-known, which required "yes" responses). Stimulus pictures (each with a viewing angle of approximately 5.37° vertically and approximately 3.58° horizontally) were presented in the center of a computer screen (1920 × 1080, refresh rate 60 Hz) with a visual distance of 80 cm from the operator. Participants were required to answer whether they knew the person in the picture by pressing a response key (for known people "F" had to be pressed, while for unknown people "J" had to be pressed) with the index finger of the left or right hand according to the confidential instructions provided by the teachers.

At the beginning of each trial, a white fixation cross was presented in the center of the black screen for 500 ms followed by a stimulus for 1500 ms. When the stimulus appeared, participants were asked to respond as quickly as possible. Overall, the experimental session involved 4 blocks of 120 trials, each of which contained 30 trials of three types of stimuli. In each block, probe stimuli (photos of the two teachers) had different images, and nonprobe stimuli were also not repeated. There was a training session before the experimental session. At the end of the survey, we debriefed the participants whether they were cognizant of the person presented in the picture and whether they had deceived to keep the secret. It was based on debriefing that the data of those who did not deceive were excluded from the analysis.

### EEG recording

The participants sat comfortably in front of the computer in a dimly lit room. The electroencephalogram (EEG) was continuously recorded using 64-channel Ag/Ag–Cl electrodes mounted in an active electrode cap (actiCAP, Brain Products, Germany) based on the 10–10 system montage. The electrodes were referenced to Cz, and the ground electrode was placed at the center of the forehead. The EEG signals were recorded using Brain Vision Recorder2 (Brain Products GmbH, Gilching, Germany) at a sampling frequency of 500 Hz and filtered with a 0.01–100 Hz bandpass filter. The impedance was kept lower than 5 kΩ. Vertical and horizontal electro-oculograms were recorded using electrodes below and on the outer canthus of the left eye and used to correct EEG for eye movement artifacts.

### Data processing and analysis

#### Data processing

According to the type of response, the trials were sorted into two categories: (1) truthful responses that were made to the nonprobe stimuli and (2) deceptive responses (Facing a person you know, but responded with a keystroke indicating that you don't know him or her) that were made to the probe stimuli. Trials that did not meet the above classification criteria were excluded. In addition, since there are two kinds of truthful responses to nonprobe stimuli (unknown and known) and one kind of deceptive response to probe stimuli (unknown), we only focused on trials in which “unknown” answers were given, and nonprobe stimuli of “known” responses that were used to eliminate prepotent response keystrokes of “unknown” were excluded. The artefact-free data included for comparative outcome analysis included 96.14 ± 30.28 epochs for deceptive responses and 110.24 ± 41.02 epochs for truthful responses (M ± SD).

#### Behavioral analysis

Response time (RT) was defined as the time interval between the appearance of the stimulus and the response key press. To eliminate the impact of outliers, reaction times more than 2.5 standard deviations from the mean per subject and condition were removed^38,72^. Comparisons of RTs between deceptive and truthful responses were performed using paired t-tests.

#### ERP analysis

**Figure 5:**
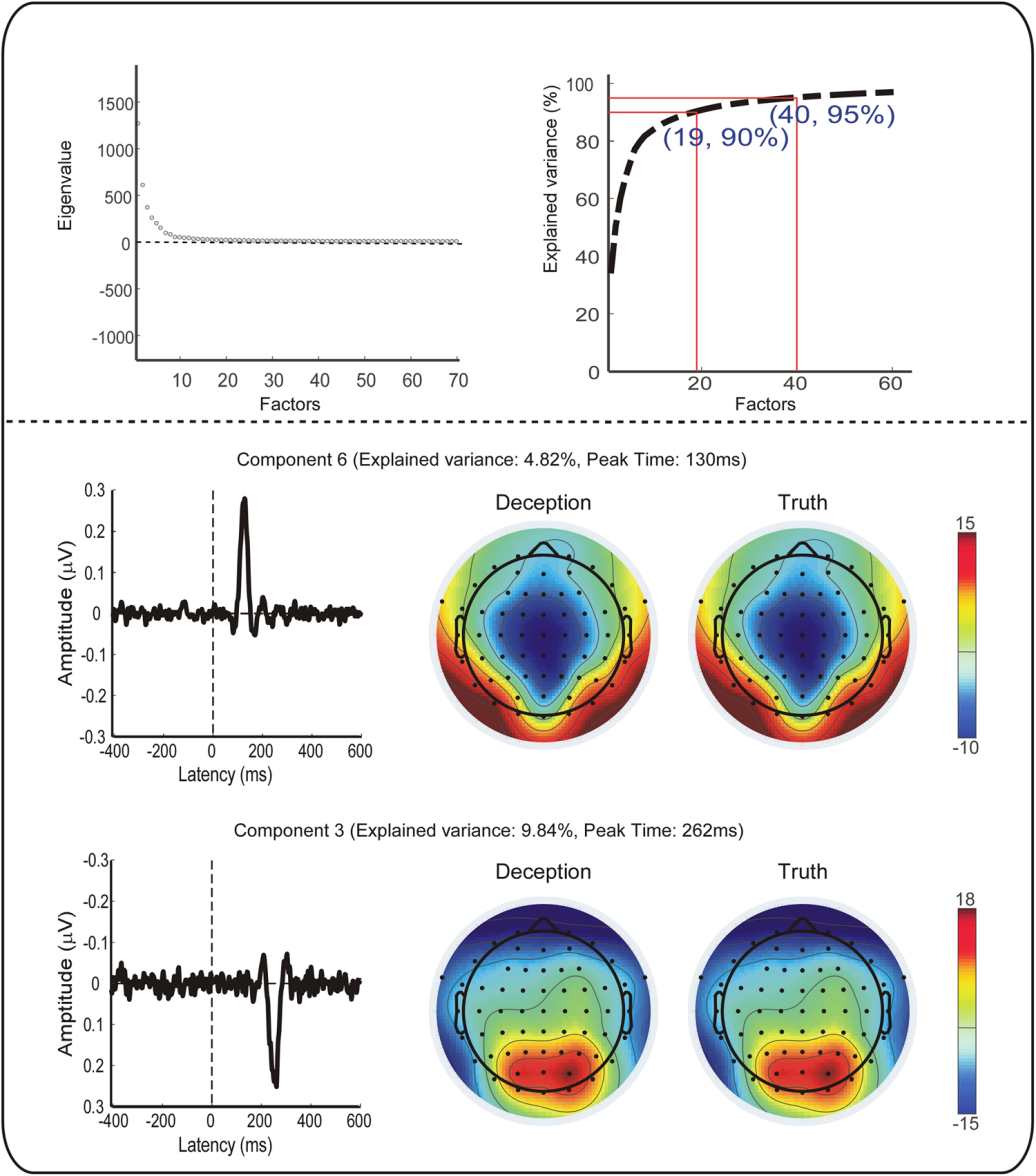
Top panel: Eigenvalue of factors and explained variance of accumulated factors. Bottom panel: extracted components for the ERPs of interest (MFN, RLP) and corresponding topography (time window: 110–150 ms, 240–280 ms). Component 6 corresponds to MFN, with an explained variance of 4.82% and a peak point of 130 ms. Component 3 corresponds to RLP, this component explained 9.84% of the variance, and its peak point is 262 ms.

ERP analysis was performed in MATLAB R2013b (The MathWorks Natick, MA, USA) and EEGLAB v12.0.2.6b. All electrodes were rereferenced to the bilateral mastoids (TP9 and TP10 electrodes), and the Cz electrode was reinstated. A bandpass filter of 0.1–30 Hz was used to filter the data. The continuous EEG was segmented into discrete epochs from 500 ms before to 800 ms after response onset. The window preceding a response in a range from 400 to 200 ms was used as the baseline^73^. Independent component analysis was performed to identify and remove ocular and prominent muscle artifacts^74^. For response-locked ERP components, the MFN was defined as the most negative peak encountered at 110–150 ms after the response onset. An obvious positive deflection was observed after MFN, which has not been previously reported in published literature. In the absence of a published name, we labeled the component response-locked positivity (RLP). RLP was determined as the maximum positive peak following MFN within a 240–280 ms postresponse interval. The peak amplitudes of the MFN and the PLR were further compared between two conditions using a paired sample t test at all scalp electrodes, an FDR correction was applied for pairwise electrode comparisons. The peak amplitude of ERP components at three midline anterior–posterior sites were further analyzed (Fz, FCz, Cz). Since MFN and RLP are subsequent ERPs, their magnitude may influence each other, and thus the condition effects on deceptive vs. truthful responses may be affected. To extract the independent MFN and RLP, temporal-principal component analysis (t-PCA) was also performed, which is a data reduction technique that helps to disentangle overlapping ERPs^75^. Specifically, the data set was formed into a matrix (time samples × (channels × conditions × subjects)) to explore the components of interest^76,77^. The temporal and spatial components of interest were simultaneously extracted by PCA and Promax rotation^78,79^, and projected to the electrode field to correct the variance and polarity indeterminacy. Then, the amplitude of the back-projected components was calculated on the electrode of interest^80,81^. The t-PCA has been performed based on covariance matrix between variables^81^. Thirty components were disentangled for the time range that has been applied to perform the grand average, which explain > 90% of the total variation (see Fig. 5). Component 6 corresponds to MFN, with an explained variance of 4.82% and a peak time of 130 ms. While component 3 corresponds to RLP, this component explained 9.84% of the variance, and its peak time is 262 ms. The standardized component loadings and scores are shown in Fig. 5. Two-way repeated measures analysis of variance (ANOVA) was performed with the within-subject factors of condition (truth, deception) and site (Fz, FCz and Cz). Greenhouse–Geisser corrections were used where the sphericity assumptions were violated. Effect sizes were measured using a partial eta-squared coefficient η^2^_p_^2,82^.

#### Source localization analysis

Standardized low-resolution brain electromagnetic tomography (sLORETA)^83^ was used to compute the underlying cortical three-dimensional distribution of the MFN and RLP. The cortical gray matter was divided into 6239 voxels with dimensions of 5 × 5 × 5 mm. The spatial electrode coordinates were defined according to the MNI atlas^84^. The current source density for each voxel of each individual for the two conditions was calculated. To localize differences in ERP activity between deception and truth conditions, paired t-tests were used on log-F-ratio data at each voxel. Statistical testing was calculated using a nonparametric randomization test (Statistical non-Parametric Mapping; SnPM). The method utilized Fisher’s random permutation test with 5000 randomizations to correct for multiple comparisons.

#### Time–frequency analysis

For each trial, time–frequency analysis was performed using MATLAB R2013b (The MathWorks Natick, MA, USA) from − 500 to 800 ms in the time domain and from 1 to 30 Hz (in steps of 0.5 Hz) in the frequency domain. A time–frequency decomposition of each epoch was performed to obtain a windowed Fourier transform (WFT) with a fixed 200-ms Hanning window. Such a window width allows us to obtain a good tradeoff between the time resolution and the frequency resolution within the explored range of frequencies^85^. The power spectral densities were baseline-corrected (− 400 to − 100 ms before the response) at each frequency. The baseline correction was obtained by dividing the baseline-subtracted values of each frequency by the average of the baseline values of that frequency^86^. Based on the point-by-point statistical analysis of the time–frequency maps at all scalp electrodes, the response-induced event-related spectral perturbations (ERSPs) were largest in the left prefrontal cortex (FP1, AF3, AF7), and previous studies have also shown such a scalp distribution^9,50^. The AF7 electrode was chosen to analyze the mean converted amplitude of the time–frequency region of interest (ROIs). A paired t-test was performed to analyze the difference in neural oscillation between the deception and truth conditions. In multiple comparisons, the false discovery rate was used to adjust the statistical test significance level^87^.

## Data Availability

The datasets generated during and/or analysed during the current study are available from the corresponding author on reasonable request.
